# Microglial Polarization: Novel Therapeutic Strategy against Ischemic Stroke

**DOI:** 10.14336/AD.2020.0701

**Published:** 2021-04-01

**Authors:** Yimeng Xue, Ding Nie, Lin-Jian Wang, Han-Cheng Qiu, Long Ma, Ming-Xin Dong, Wen-Jun Tu, Jizong Zhao

**Affiliations:** ^1^Department of Neurosurgery, Beijing Tiantan Hospital, Capital Medical University, Beijing, China.; ^2^Savaid Medical School, University of Chinese Academy of Sciences, Beijing, China.; ^3^Institute of Radiation Medicine, Chinese Academy of Medical Science & Peking Union Medical College, Tianjin, China.; ^4^China National Clinical Research Center for Neurological Diseases, Beijing, China.; ^5^Center of Stroke, Beijing Institute for Brain Disorders, Beijing, China.; ^6^Beijing Key Laboratory of Translational Medicine for Cerebrovascular Disease, Beijing, China

**Keywords:** microglia, cell polarization, ischemic stroke, mechanism, treatment

## Abstract

Ischemic stroke, which is the second highest cause of death and the leading cause of disability, represents ~71% of all strokes globally. Some studies have found that the key elements of the pathobiology of stroke is immunity and inflammation. Microglia are the first line of defense in the nervous system. After stroke, the activated microglia become a double-edged sword, with distinct phenotypic changes to the deleterious M1 types and neuroprotective M2 types. Therefore, ways to promote microglial polarization toward M2 phenotype after stroke have become the focus of attention in recent years. In this review, we discuss the process of microglial polarization, summarize the alternation of signaling pathways and epigenetic regulation that control microglial polarization in ischemic stroke, aiming to find the potential mechanisms by which microglia can be transformed into the M2 polarized phenotype.

## 1. Introduction

Stroke is the second leading cause of death and the highest disabling disease in the world, with an increasing incidence in developing countries [1-3]. China suffers the greatest burden of stroke globally, with about 2.4 million new cases and 1.1 million stroke-related deaths annually [4]. Ischemic stroke induced by arterial occlusion is the major cause of strokes and account for ~71% of all strokes in the world. The standard treatment for acute ischemic stroke is intravenous thrombolysis with tissue-type plasminogen activator (t-PA) and endovascular treatment which are time-critical [5]. A nationwide population-based study reported that only approximately 20% of stroke patients received thrombolytic therapy within 3 hours in China [6]. Therefore, it is urgently needed to establish other potential therapies.

Immunity and inflammation play an important role in the pathophysiology of stroke [7]. Being the key innate immune cells, microglia act as guardians responding to various acute brain injuries, including ischemic stroke[8, 9]. As the resident macrophages of the central nervous system (CNS), the morphology and gene expression of microglia change while responding to brain injury, such is called microglial activation [10]. Activated microglia is one of the most important cellular components of poststroke neuroinflammation, which occurs within an hour to more than a month, developing four morphological states: ramified, intermediate, amoeboid and round [11-13]. Age is a critical co-factor for CNS diseases. Interestingly, the function of microglial cells changed with aging and the morphology of the microglia is more de-ramified [14]. Compared with young microglial cells, aged microglia activation is amplified and prolonged [15]. The existence of an aging-related microglial phenotype in the aged human brain is verified and it is involved in pathological processes of CNS diseases [16].

Microglia could present different phenotypes in accordance with the stimulus, the environment, and the period, which is called microglial polarization [17, 18]. Similar to macrophages, microglial polarization is divided into classically activated (M1, pro-inflammatory) phenotype and alternatively activated (M2, anti-inflammatory) phenotype. Many differences of the polarization of these two cell types have been noted [19]. Polarized microglia differ from polarized macrophages in protein expression, phagocytosis, and injury response. In response to inflammatory factor, M2 microglia are more protective and tend to maintain the M2 phenotype status [20]. The phenotype of microglial cells also changed with aging. Aged microglia demonstrated a propensity for the development of a pro-inflammatory phenotype with increased pro-inflammatory cytokines and inflammatory receptors, which is referred to as primed, reactive or sensitized [15]. Microglia play an important role in various neurological diseases, involving in multiple aspects of neuroinflammation, such as cytotoxicity, repair, immunosuppression and regeneration at the basis of different polarization states [21]. Thus, we make a review to discuss the process of microglial polarization and summarize the alternation of signaling pathways and epigenetic modifications that control microglial polarization in ischemic stroke, aiming to find the potential mechanisms and drugs by which microglia can be shifted from M1 into the M2 polarized type after ischemic stroke.

**Table 1:** Characteristics of M1 and M2 microglia.

	Stimulus	Phenotypic markers	Substances produce	Function
M1	IFN-γ, LPS	iNOS, TNF-α, MHCII, CD86	IL-23, IL-18, IL-12, IL-1β, IL-6, TNF-α, NO, CCL2, CXCL10, ROS, MMP9, MMP3	Proinflammatory, Phagocytosis, Cytotoxicity, Present antigens, Kill intracellular pathogens
M2a	IL-4, IL-13	Arg-1, Fizz-1, Chitinase3-like 3, Chemokines IGF-1, CD206,	Extracellular matrix proteins	Tissue repair; Remodeling of extracellular matrix; Phagocytosis
M2b	Immune complex, TLRs agonists	IL-10, Cyclooxyge- nase 2, Sphingosine kinase, suppressor of cytokine signaling 3	IL-1β, IL-6, IL-10, TNF-a	Phagocytosis Removal of tissue debris
M2c	IL-10, TGF-β, glucocorticoid	CD163	IL-10, TGF-β	Anti-inflammatory Phagocytosis

## 2. Microglial Polarization

Microglia act as a ‘double-edged sword’ in the CNS by representing neurotoxic or neuroprotective functions according to phenotypic polarization [22]. M1 polarized microglia secrete inflammatory cytokines that lead to tissue damage. In contrast, M2 microglia have a neuroprotective effect by producing anti-inflammatory cytokines, inhibiting nerve injury, and promoting tissue repair [23]. M1 microglia are characterized by its amoeboid shape, high mobility, producing various pro-inflammatory cytokines. Interferon γ (IFN γ) secreted by T helper 1 cells activates signal transducer and activator of transcription 1 (STAT1) factor through Janus kinase (JAK)1/JAK2 signaling, inducing M1 microglia to produce pro-inflammatory cytokines [24]. Another pathway is activited by lipopolysaccharide (LPS) or damage-associated molecular pattern (DAMP) stimulation accompanied with Toll-like receptor 4 (TLR4) [25, 26]. Along with producing various pro-inflammatory cytokines (IL-23, IL-18, IL-12, IL-1β, IL-6, TNF-α, CCL2, and CXCL10), ROS, NO, and proteolytic enzymes matrix metalloproteinase-9 (MMP9) and matrix metalloproteinase-3(MMP3) [27-30], M1 microglia serve as antigen presentation to avoid pathogens invasion [31].

Alternatively, activated M2 microglia are composed of three subtypes with unique markers and biological function: M2a, M2b and M2c [32]. With the stimulation of IL-4, IL-13, M2a microglia display enhanced expression of Arginase-1, Ym1, Insulin-Like growth Factor-1 (IGF-1), CD206, chitinase 3-like 3 and found in the inflammatory zone1 (Fizz1) [33, 34], mainly contributing to cell regeneration. Induced by immune complexes and TLRs agonists, M2b phenotype produce increased expression of IL- 1β, CD86, suppressor of cytokine signaling 3 (SOCS3), IL-1β, IL-6, IL-10, involving in phagocytosis and removal of tissue debris [35, 36]. When the response of inflammatory shows weakened, transforming growth factor β (TGF-β), IL-10, and glucocorticoids induce M2c phenotype to help tissue regeneration [37] (see Table 1).

In response to an immune challenge, the process of microglial polarization shift towards priming with aging [38]. Under the stimulation of LPS, aged microglia showed hyperactive response with higher induction of inflammatory IL-1 and anti-inflammatory IL-10. And aged microglia prolonged the downregulation of the fractalkine receptor and failed to up-regulation of IL-4 receptor[39]. Taken together, the ability of microglia to lower inflammation in the brain is impaired. The understanding of the aged microglia phenotype and function in humans is limited, particularly in the process of microglial polarization. Mounting evidence is needed to confirm the role of aged microglia polarization in ischemia stroke.

## 3. Microglial Polarization in Neurological Disorders

Although it is oversimplified to divide microglia into the M1 and M2 phenotypes, the classification has important implications for comprehending the role of microglia in CNS diseases [40]. The role of microglial polarization in a variety of neurological disorders has been illuminated. Targeting M2 phenotype polarization has been proved to be a potential therapeutic strategy. In Alzheimer’s disease (AD), studies have shown that the dysfunction of M2 microglia and the excessive activation of M1 microglia promote inflammatory pathological injury. Through polarization moderation, microglia could induce tissue repair and phagocytosis to reduce Aβ levels, alleviating AD pathological damage [22]. In AD mouse models, DSP-8658 and Bexarotene have proved to enhance microglial Aβ phagocytosis[41, 42]. In Parkinson’s disease (PD), the dopaminergic degeneration is involved in microglial polarization, Rosiglitazone boosts the M2 phenotype over the pro-inflammatory phenotype modulating microglia polarization [43]. Although the pathology of amyotrophic lateral sclerosis (ALS) has still not been completely understood [44], hirsutella sinensis prolongs the lifespan of ALS mice by promoting transition of microglial polarization from M1 to M2 phenotype[45]. In Huntington’s disease, microglial polarization affects striatal neuronal dysfunction [46]. In multiple sclerosis (MS), M1 microglia have a greater ability to present antigens, leading to demyelination and neurodegeneration, while M2 microglia protect oligodendrocytes and neurons from damage and ameliorate disease severity[26]. A recent clinical trial showed that anti-pathogenic human endogenous retrovirus type W (pHERVW) envelope protein (ENV)-mediated microglial polarization exerts neuroprotective effects in MS[47] (see Table 2).

**Table 2:** Summary of microglial polarization in neurological disorders.

Neurological disorders	The function of polarized microglia	Model	Drugs (M2→M1)
Alzheimer’s disease (AD)	M1 phenotypic inhibits Aβ clearance, while M2 phenotypic enhances Aβ clearance.	Mouse model	DSP-8658 Bexarotene
Parkinson’s disease (PD)	Dopaminergic degeneration is associated with microglial polarization.	Mouse model	Rosiglitazone
Amyotrophic lateral sclerosis (ALS)	Elimination of apoptotic cells, production of growth factors, maintenance of synapse structure and function are the main function of microglia.	Mouse model	Minocycline Rho kinase inhibitor[114] Hirsutella sinensis
Huntington’s disease (HD)	Microglial polarization affects striatal neuronal dysfunction.	Mouse model	Minocycline
Multiple Sclerosis (MS)	M1 microglia have a greater antigen presenting ability, leading to demyelination and neurodegeneration. While M2 microglia protect oligodendrocytes and neurons from damage and ameliorate disease severity.	Clinical phase IIb	Anti-pathogenic human endogenous retrovirus type W envelope protein (pHERV-W ENV)
Neurological disorders	The function of polarized microglia	Model	Drugs (M2→M1)

## 4. Polarized Microglia-based Therapy in Ischemic Stroke

While ischemic stroke occurs, the microenvironment of microglia has changed and classic (M1) or alternative (M2) microglia are polarized responding to peripheral inflammation. At the early stage of ischemic stroke, microglia tend to assume the M2 phenotype responding to acute injury, and then microglia transform into the M1 phenotype that induces an inflammatory response [48]. The mechanism of microglial polarization during ischemic stroke involves multiple pathways that have not been entirely clear. Present studies showed that the type of microglial polarization was decided by signaling pathways. Understanding the accurate mechanism of microglial polarization, we can find a breakthrough in the treatment. In the following, we discuss the transcription factors and epigenetic regulation associated with ischemia-induced microglial polarization to find out the mechanism of microglial M1 to M2 transition (see Table 3).

**Table 3:** Studies of polarized microglia-based therapy in ischemic stroke.

Drug/agent	Model	Mechanism	Effect	Reference
TWS119	MCAO mice	Wnt/β-catenin pathway activator	Modulate microglia to anti-inflammatory phenotype	[115]
Melatonin	MCAO mice BV2 microglia	STAT3 pathway activator	Decrease expression of pro-inflammatory markers and increased expression of anti-inflammatory markers	[116]
HAMI3379	Rat	CysLTR antagonist NF-κB pathway	Inhibit microglia M1 polarization and promote microglia polarization toward M2 phenotype	[117]
β-caryophyllene (BCP)	MCAO Mice	TLR4 pathway antagonist	Decrease the secretion of pro-inflammatory cytokines (IL-1β, TNF-α) and polarize microglia towards the M2 phenotype	[118]
Suberoylanilide hydroxamic acid	MCAO mouse	Histone deacetylase inhibitors	Suppresse M1 cytokine expression (IL-6, TNF-α, and iNOS) while promoted the transcription of M2 cytokines (Arg-1 and IL-10)	[119]
Isosteviol Sodium (STV-Na)	MCAO mouse BV2 microglia	miR-146a-5p	Promote M2 polarization and inhibit M1 response	[120]
Baicalein	MCAO rat	NF-κB antagonist	Reduced expression of the M1 marker (CD 16 and CD86), and increase expression of the M2 marker, (CD 163 and CD206)	[121]
Berberine	MCAO mice	AMPK activator	Inhibit M1 polarization and promote M2 polarization	[122]
CKLF1	MCAO mice	NF-κB activator	Modulated primary microglia skew toward M1 phenotype	[123]
Exosomes from LPS-stimulated macrophages	Rat		Skew the microglial functional polarity from M1 toward an anti-inflammatory M2 phenotype.	[124]
Nicotinamide phosphoribosyltransferase (NAMPT)	MCAO mice		Inhibite pro-inflammatory *microglia*, promoted *microglia polarization* toward the anti-inflammatory phenotype,	[125]
Propagermanium	MCAO mice	CCR2 inhibitor	Inhibite inflammatory cytokines releasing, such as TNF-α, IFN-γ, IL-1β, IL-6, IL-12, IL-17, and IL-23, inhibite CD16 expressed in *microglia*.	[126]
Glycine	SpragueDawley rats BV-2 cells	NF-κB p65 inhibitor	Inhibite M1 microglial polarization	[127]
Xuesaitong	MCAO mice	STAT3 inhibitor	Promote the *polarization* of *microglia* to an M2 phenotype	[128]
Sphingosine 1-phosphate receptor subtype 3 (S1P)	MCAO mice	MAPK and Akt activator	Involve its modulation of microglial activation and M1 *polarization*	[129]
L-3-n-Butylphthalide	MCAO mice		Skewing M1 *microglia polarization* towards M2	[130]
α-Lipoic acid	MCAO rat	NF-κB inhibitor	Induced the *polarization* of *microglia* to the M2 phenotype, modulated the expression of IL-1β, IL-6, TNF-α and IL-10,	[131]
Hypothermia	MCAO mice		Reduce the number of CD16-positive M1 *microglia* and increase the numbers of CD206-positive M2 *microglia*	[132]
Ischemic postconditioning	Rat		Polarize to a ramified morphology with higher expression of M2-like markers	[133]
XQ-1H	MCAO mice BV2 *microglia*	PPARγ pathway activator	Regulate microglia polarized from pro-inflammatory into anti-inflammatory phenotype	[134]
Salidroside	MCAO mice		Reduce the expression of M1 *microglia* markers and increased the expression of M2 *microglia*	[135]
Anisalcohol	BV2 *microglia*	NF-κB inhibitor MAPK activation	Down-regulated the expression of the M1 marker CD16/32 and up-regulated that of the M2 marker CD206.	[136]
Fas ligand incapacitation	Mouse	NF-κB pathway	Alleviate CD4 T cells-induced inflammation induce M1 *microglia polarization*	[137]
CD8 receptor	MCAO rat	CD8 signaling	Repolarize IL4-treated M2 cells to an M1 phenotype	[138]
Hyperforin	Mice		Shift from M1 to M2 phenotypes	[139]
Apoptosis signal-regulating kinase 1	BV2 microglia		Control the polarization of M1/M2	[140]
Erythropoietin	MCAO mice		Reduce M1 *microglia* and increase M2 *microglia*	[141]
Curcumin	MCAO mice		Promot M2 microglial *polarization* and inhibite *microglia*-mediated pro-inflammatory responses	[142]
Hydrogen sulfide	MCAO mice	AMPK Pathway activation	Promoted a shift from pro-inflammatory phenotypes toward anti-inflammatory phenotypes in microglial *polarization*.	[143]
HP-1c	Mice	AMPK-Nrf2 pathway activation	Shift the M1/M2 *polarization*	[144]
Progesterone	Rat		Modulate polarized *microglia*	[145]
Long noncoding RNA H19	MCAO mice BV2 microglia		HDAC -dependent M1 microglial *polarization*	[110]
Lipoxin A	MCAO rat		Increase anti-inflammatory M2 *microglia*	[146]
Thiamet G	MCAO mice BV2 *microglia*	NF-κB inhibitor	Decrease expression of the M1 markers, and increase expression of the M2 markers	[147]

### 4.1 Transcription Factor

Two important transcription factors, c-AMP response element binding protein (CREB) and nuclear factor-κB (NF-κB), are involved in the mechanism of microglial polarization[49]. NF-κB is a traditional transcription factor activated by LPS and expressed in many cell types in the nervous system [50, 51]. There are five members of the NF-κB family, including NF-κB1 (p50), NF-κB2 (p52), RelA (p65), RelB and c-Rel. More pieces of evidence suggest that NF-κB signaling plays an important role in inflammatory diseases and has biphasic functions in ischemic stroke [51-53]. NF-κB signal pathway is related to the expression of M1 phenotype genes (IL-1, IL-2, IL-6, IL-12, TNF-α, inducible nitric oxide synthase (iNOS), and cyclooxygenase-2 (COX-2)), playing a detrimental role in ischemic stroke[50, 54]. The expression of matrix metalloproteinases (MMPs) is mediated by NF-κB signaling, leading to blood brain barrier damage and brain inflammatory cell infiltration [55, 56]. CD147 (cluster of differentiation 147) could induce extracellular MMP, being a promising therapeutic target for ischemic stroke [56]. In contrast, NF-κB p50 is a key redox signaling mechanism regulating the M1/M2 balance in microglia. NF-κB p50 homodimers could play a negative role in STAT1 activity and M1 phenotype gene transcription, increasing M2 polarized mediators (Arg-1, Ym1 and Fizz1) [57]. Lower NF-κB p65 expression has potential protective effect by promoting M2 phenotype microglial polarization and alleviating inflammation[58]. Other transcription factors may regulate microglial polarization by influencing the activity of NF-κB. Notch signaling promote production of IFN-γ through recruitment of p50 and c-Rel, in response to LPS. With NF-κB activation, inflammation and neurotoxicity exacerbate ischemic brain damage [59]. The crosstalk between Notch and NF-κB inhibits the expression of PPARγ which is necessary for the induction of the M2 phenotype [60, 61]. STAT1 and STAT3 are able to increase the expression of NF-κB p65. Inhibiting the activation of STAT1 and STAT3 prevents the inflammatory reaction caused by brain ischemia, thereby reducing the occurrence of infarction and edema.

In contrast, CREB cooperated with C/EBPβ promote tissue repair by amplification of M2-specific gene [62]. Confoundingly, the expression of M1-specific genes associated with inflammation is also affected by C/EBPβ [63]. The role of C/EBPβ in regulating microglial phenotypes depends on the competitiveness of CREB and NF-κB[64]. CREB-binding protein (CBP) is another competition site. The increase of CREB activity has a negative effect on the combination of CBP and NF-κB [65, 66]. With the activation of TLRs, interferon regulatory factor-3 (IRF-3) is phosphorylated and interacts with CBP promoting the M2 polarization. The RelA/CBP/p300 complex is formed at the same time [67-69]. In summary, the balance of NF-κB and CREB plays a crucial role in the microglial polarization in cerebral ischemia [49].

In addition, nuclear factor erythroid 2-related factor 2 (Nrf2) is activated and involved in the anti-inflammatory effect of the M2 phenotype microglia, which is a key factor of brain endogenous defense system, in response to oxidative stress [70, 71]. After the activation of Nrf2, neuro-inflammation induced by LPS was inhibited both *in vivo* and *in vitro* [72, 73]. A study concluded that achyranthes bidentata polypeptidek's could inhibit neuro-inflammation in BV2 microglia through Nrf2 dependent mechanism[74]. Through the activation of the Nrf2 pathway and the inhibition of the NF-κB pathway, Biochanin A may contribute to the neuro-protection against ischemic injury in rats by anti-oxidative and anti-inflammatory actions [75]. Other studies conclude that the disruption of mTORC1 pathway could shift microglial phenotype to decrease brain inflammation [76].

### 4.2 Epigenetic Modifications

Besides the transcription factors above, the polarization and functional status of microglia require precise regulation of target gene expression, which can be achieved by epigenetic modifications. Epigenetics refers to modifications that do not alter the genetic code but control how information is encoded in DNA in a tissue- and context-specific manner developmentally or environmentally [77]. The mechanisms of epigenetic modifications are usually mediated by modifications of histones and other chromatin proteins (such as methylation, acetylation, and phosphorylation), methylation of CpG DNA motifs, hydroxymethylation, and non-coding RNA [78, 79]. The epigenetic markers histone modification and miRNA involved in microglial polarization and activation processes are reportedly more than the others [80]. The following summarizes the recent findings on the role of epigenetic modifications regulating microglial polarization.

#### 4.2.1 MiRNA

MicroRNAs (miRNAs) are small non-coding RNA molecules that regulate gene expression post-transcriptionally. MiRNAs repress gene expression by combining with the 3’-untranslated region, coding sequence, or 5’UTR of target genes [81, 82]. A total of 30%-90% of human genes are regulated by miRNAs that modulate cell growth, activation, and differentiation [83]. M1- and M2-polarized microglia exhibit distinct miRNA profiles. Recent research has also defined a role for miRNA in microglial polarization [84]. With the development of miRNA research, more and more miRNAs are related to microglial polarization phenotypes.

It is well accepted that miRNA-155 expression promotes M1 polarization by suppressing M2-signature genes and that miRNA-124 enhances the M2 phenotype by targeting M1 genes [85-87]. In MACO mice, miR-124 proved to increase the survival of neuron and M2 microglial polarization [81]. In IL-4 stimulated microglia, miR-145 was the most increased miRNA, facilitating the M2 phenotype in microglia[88]. Overexpression of miR-146a contributed to polarization transitions from M1 to M2 phenotype in microglia [89]. Isosteviol sodium can downregulate miRNA-181b to protect mouse brain with ischemia stroke by repressing NF-κB signaling pathways, providing a novel therapy for ischemic stroke [90]. MiRNA-128 could reduce the M1 phenotypic markers and increase the M2 phenotypic markers, promoting the viability of microglia [91]. Overall, targeting pro-inflammatory or anti-inflammatory miRNAs to regulate the microglial polarization provides new direction in the treatment of ischemic stroke. However, further studies are badly in need to clarify the function of miRNAs in the switch of microglial phenotype. Additionally, how to deliver miRNAs to the central nervous system (CNS) through the blood brain barrier (BBB) and prevent the degradation of miRNAs are also unsolved. With mechanisms of microglial polarization unveiled, targeting specific miRNAs may provide major restorative therapies and microglial polarization-based therapy will be potential future research field of the treatment of ischemia stroke [92].

#### 4.2.2 DNA Methylation

DNA methylation is an epigenetic process catalyzed by DNA methyltransferases (DNMTs). Methyl groups are added to DNA nucleotides, which leads to chromatin condensation and alteration of gene expression [93]. DNMT maintains cytosine methylation through mitotic and meiotic cell divisions and is widely expressed in brain tissue. The whole DNA methylation in brain is up-regulated after cerebral ischemia, which may control gene expression profile in cerebral ischemia injury [94, 95]. Aberrant DNA methylation patterns have been proved in cerebral ischemia. Reduced DNA methylation play a neuroprotective role in ischemic stroke. Inhibition of DNMT1 expression affects chromatin structure and increases expression and combination of transcription factors (such as hypoxia-inducible factor-1 (HIF-1)) with neuroprotective genes [96, 97]. It has been reported that there is an intrinsic link between DNA methylation in microglia and aging-mediated cognitive deficits [98]. However, the role of DNA methylation has remained to be further elucidated in microglial polarization in ischemic stroke. DNA methylation is a modifiable regulation and it is possible that in the future methylated or unmethylated genes could be a drug target for stroke treatment.

#### 4.2.3 Histone Modifications

**Figure 1.**
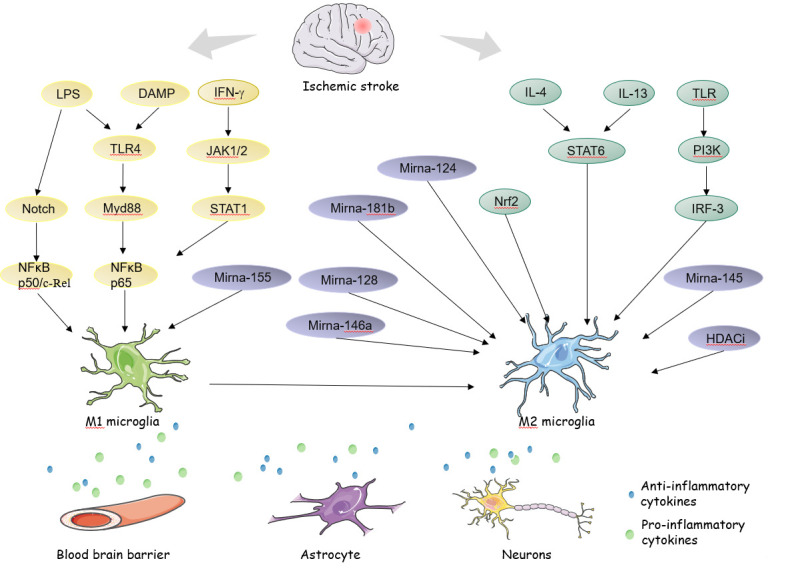
Microglia polarization after ischemic stroke. M1 microglia produce pro-inflammatory cytokines to exacerbate neural death, astrocyte apoptosis, and blood brain barrier (BBB) disruption. Conversely, M2 microglia produce anti-inflammatory cytokines to maintain BBB integrity, promote the proliferation and differentiation of neural cells and tissue repair.

The electrostatic interaction of positive charges on histones and negative charges on DNA inhibits tightly packed chromatin structures[96]. The acetylation of histones on lysine residues can neutralize the positive charge, thereby disrupting the stability of the histone-DNA interaction, and subsequently changing the concentrated chromatin into an open, loosely packed chromatin structure, allowing gene recruitment activators or inhibitors of transcription and it can be reversed by histone deacetylases (HDACs) activity [99, 100]. It has been reported that HDAC inhibitors (HDACi) have anti-inflammatory effects in neuroprotection [101]. The protection of HDACi on microglia polarization is involved in its anti-inflammatory effect in the early phase of cerebral ischemia, reducing the activation of microglia and promote activated microglia to protective phenotype, providing a promising therapeutic intervention [102, 103]. It has been reported that the inhibition of HDAC1 and HDAC2 activity after transient cerebral ischemia promotes microglia polarization towards M2 Phenotype[104]. Valproic acid treatment attenuated the inflammatory response by modulating microglia polarization through STAT1-mediated acetylation of the NF-κB pathway, dependent of HDAC3 activity [105]. Enhancer of zeste homolog-2 (EZH2), a histone methyltransferase, has been recognized to promote M1 microglial polarization but repress M2 microglial polarization probably via activating STAT3[106]. On the contrary, histone 3 lysine 27 (H3K27) demethylase Jumonji d3 (Jmjd3) promotes M2 microglial polarization but represses M1 microglia polarization [107, 108]. Dehydroepiandrosterone (DHEA) is the most abundant circulating steroid hormone in humans, TrkA signaling activated by DHEA is an effective regulator of inflammation through Jmjd3-dependent pathway, providing potential treatments for neuroinflammatory diseases (Fig. 1) [109].

Besides above, there are other epigenetic regulations contributing to the polarization of microglia in the ischemic stroke. Long noncoding RNA H19 promotes neuroinflammation by driving HDAC1-dependent M1 microglial polarization, suggesting a novel H19-based diagnosis and therapy for ischemic stroke [110]. MiR-30d-5p- enhanced adipose-derived stem cells (ADSC) derived exosomes prevent cerebral injury by inhibiting microglial polarization to M1 [111]. Investigation of epigenetic regulation of microglia polarization and function is at an early stage and there are many unknown areas for future research. Finally, recent breakthroughs have opened a new door to epigenetic therapy of ischemic stroke.

More and more evidence has revealed that modulators of microglial phenotypes may be a promising therapeutic approach for the treatment of ischemic stroke. However, fundamental differences of the cellular environment and damage-response between macrophages and microglia exist, the M1/M2 oversimple classification may not be applicable to microglia. Unbiased methods such as genome-wide transcriptomics, epigenomics and proteomics are urgent needed to aid research progress [112].

Comprehensive single-cell RNA analysis of CNS immune cells identified disease-associated microglia (DAM), which is a kind of microglia with specifically transcription and function. The emergence of DAM may provide a new explanation for the contradictory views on the detrimental or beneficial effects of microglia in recent years [113].

## 5. Conclusion

In cerebral ischemia, the neuroprotective effects of M2-polarized microglia cells include clearing debris as well as promoting tissue repair. Increasing evidence indicates that shifting microglial phenotype from the pro-inflammatory M1 state toward the anti-inflammatory M2 phenotype may be an effective therapeutic strategy for ischemic stroke. Importantly, several signalling pathways—such as NF-κB, and Wnt/β-catenin—may be critically involved in microglial polarization in ischemic stroke. The underlying mechanisms of microglial polarization in ischemic stroke are still not well understood and need to be further elucidated.
